# Mechanistic Evidence for Hg Removal from Wastewater by Biologically Produced Sulfur

**DOI:** 10.3390/toxics12040278

**Published:** 2024-04-10

**Authors:** Seok-Soon Jeong, Byung-Jun Park, Jung-Hwan Yoon, Mary Beth Kirkham, Jae-E. Yang, Hyuck-Soo Kim

**Affiliations:** 1 Department of Biological Environment, Kangwon National University, Chuncheon 24341, Republic of Korea; jssddg888@naver.com (S.-S.J.); phkq26@kangwon.ac.kr (B.-J.P.); yoonfnfg@hanmail.net (J.-H.Y.); yangjay@kangwon.ac.kr (J.-E.Y.); 2 Department of Agronomy, Kansas State University, Manhattan, KS 66506, USA; mbk@ksu.edu

**Keywords:** biologically produced sulfur, mercury removal, adsorption, kinetics, mechanism

## Abstract

A significant quantity of biologically produced sulfur (BPS) is generated as a by-product of chemical and biological desulfurization processes applied to landfill gas treatment. The beneficial upcycling of BPS has seen limited use in the environmental context. The effectiveness and underlying mechanism of BPS as an adsorbent for removing Hg^2+^ from both solution and wastewater were elucidated based on experiments encompassing surface characterization, adsorption isotherms, kinetics, and thermodynamics. The BPS exhibited remarkable efficacy in removing Hg^2+^ from solution, with the Langmuir model accurately describing the adsorption process and showing a maximum adsorption capacity of 244 mg g^−1^. Surface analysis through X-ray photoelectron spectroscopy and scanning electron microscopy revealed that Hg^2+^ complexed with sulfide on BPS surfaces, forming stable HgS. The adsorbed Hg was strongly retained in BPS, with less than 0.2% of the adsorbed Hg desorbed by strong acids. Adsorption kinetics followed the double-exponential first-order model, showing an initial rapid adsorption phase wherein 75% of the initial Hg^2+^ was removed within 5 min, followed by a slower adsorption rate. The thermodynamic parameters suggested that adsorption of Hg^2+^ by BPS was a spontaneous and endothermic process. Additionally, BPS effectively removed Hg^2+^ from wastewater, showing preference for Hg over other co-existing metals. These findings underscore the potential of BPS as an effective adsorbent for Hg^2+^ removal from wastewater.

## 1. Introduction

Mercury (Hg) pollution poses a significant threat to environmental quality and public health globally. The World Health Organization (WHO) considers Hg as one of the ten most harmful elements to public health [1]. Exposure to Hg can lead to various human health problems, including issues with child growth during pregnancy or childhood. It impacts the digestive and immune systems [2,3], as well as functions of organs such as the lungs, kidneys, and skin [4,5]. In recognition of this critical issue, the United Nations Minamata Convention on Mercury (MCM) came into effect on 16 August 2017 with the primary goal of reducing emissions (air) and discharge (water) of anthropogenic Hg and aiming to protect the environment and human health. However, Hg continues to be released into the environment from various sources, including the battery and paint industries, metal mining, and the chlorine-alkali and pesticide sectors [6,7].

Various technologies have been implemented to prevent Hg pollution in wastewater, such as adsorption, membrane separation, flotation, electro-chemical treatment, and ion exchange [8,9,10,11]. Among these, adsorption technology stands out as the most widely adopted method for effectively removing Hg from water [12]. For instance, activated carbon prepared from rice husks exhibited a maximum Hg removal capacity of 55.87 mg g^−1^ through an adsorption process, and this was attributed to its favorable pore structure and oxygen-containing functional groups [13].

According to the hard and soft acids and bases (HSAB) theory, metals can be classified as Lewis acids, which accept electron pairs from ligands. Ligands are electron donors and anions that are classified as Lewis bases. Generally, a hard electron pair acceptor (hard acid) prefers to form a complex with a hard donor (hard base). Inorganic Hg acts as a soft acceptor and, thus, can strongly interact with soft donors such as sulfur-containing compounds, including sulfides, thiols, thiourea, and thioether groups [14,15,16]. In efforts to enhance Hg adsorption capacity, researchers have attempted to modify adsorbents with S-containing compounds [16,17,18].

The practical application of using adsorbents in wastewater treatment is, however, limited due to their high cost and the requirement for a large quantity of them [19,20]. Recently, there has been growing interest in finding alternative adsorbents that are readily available and cost-effective, including adsorbents derived from natural materials or industrial by-products [20,21].

Hydrogen sulfide (H_2_S) gas emitted from landfill sites causes many detrimental problems, such as equipment damage, environmental toxicity, and odor pollution [22]. To mitigate these issues, chemical and biological desulfurization technology has been successfully adopted for the removal of H_2_S [23]. During the desulfurization process, H_2_S gas is converted into a solid, biologically produced sulfur (BPS), which is primarily composed of elemental sulfur (S_8_) with a small amount of sulfides [23,24,25]. This conversion occurs via a chemical reaction using NaOH (see Equation (1)) and biological oxidation (see Equation (2)) with *Thiobacilli* [23]. A significant quantity of BPS, exceeding 10,000 tons per year, is generated from landfill sites in Seoul, Korea. Hence, there is an urgent need for the efficient and sustainable recycling of BPS in an environmentally friendly manner.

H_2_S + NaOH → NaHS + H_2_O (Chemical conversion) (1)

NaHS + ½O_2_ → ⅛S_8_ + NaOH (Biological oxidation with *Thiobacilli*) (2)

According to the HSAB theory, it is anticipated that the sulfur-containing BPS will selectively form complexes with Hg in wastewater, demonstrating significant potential as a cost-effective and environmentally sound adsorbent for Hg, because it is a by-product of the landfill desulfurization process. In this study, BPS was evaluated as an adsorbent for removing Hg^2+^ from both aqueous solutions and wastewater. The efficacy and underlying mechanisms of using BPS for this application were elucidated through experiments involving surface characterization, adsorption isotherms, kinetics, and thermodynamics.

## 2. Materials and Methods

### 2.1. Characterization of BPS

BPS samples were obtained in slurry form from the landfill gas desulfurization plant located at the Seoul landfill site (Eco-Bio Holdings Co., Ltd., Seoul, Republic of Korea), then centrifuged to separate the solid BPS. The solid was subsequently oven-dried at 65 °C for 24 h and ground to pass through a 1 mm sieve. The chemical and mineralogical composition of BPS was determined using an X-ray fluorescence spectrometer (XRF, ZSX Primus II, Rigaku, Japan) and an X-ray diffractometer (XRD, X’Pert PRO MPD, PANalytical, Almelo, The Netherlands) with Cu Kα radiation operating at 40 kV and 250 mA, respectively. The size classes and surface areas of BPS were determined using the laser particle size analyzer (Mastersizer 3000, Malvern Instruments Ltd., Worcestershire, UK). The specific surface area of BPS was determined from N_2_ adsorption isotherms at 77 K using a Brunauer Emmett Teller (BET) Analyzer (BELSORP MAX X, MicrotracBEL, Osaka, Japan). The point of zero charge of BPS was determined using 0.1 M NaCl at pH 2.0–11.0. The pH values were adjusted using HCl and NH_4_OH. The BPS was stirred for 24 h with 0.1 M NaCl solutions of different pH values. After 24 h, the pH of resulting solution was measured after equilibrium. The difference in the initial and final pH (ΔpH) was calculated, and the pH value where ΔpH was zero indicated the point of zero charge of BPS.

### 2.2. Hg Removal Efficiency

The performance of BPS in removing Hg^2+^ was evaluated through batch experiments. A stock solution containing Hg^2+^ was prepared by dissolving 99.5% mercury chloride (HgCl_2_, Daejung Chemical, Seoul, Korea) in deionized water and diluting it to the required initial concentration before use. Table 1 presents the conditions under which batch experiments were conducted, including different pH levels, reacting times, temperatures, and Hg concentrations. Each batch of BPS and Hg solution under a specific condition was equilibrated for 24 h in a mechanical shaker (150 rpm) at 298 K, then filtered through a 0.45 µm membrane filter. The concentration of Hg in the filtrate was determined using a cold-vapor Hg analyzer (Hydra II AA, Teledyne Leeman Labs, Hudson, NH, USA).

The removal efficiency and capacity of BPS for Hg^2+^ at equilibrium were calculated using the following Equations (3) and (4), respectively.

(3) $$R=\frac{\left(C_{0}-C_{e}\right)}{C_{0}}\times 100$$

(4) $$q_{e}=\frac{\left(C_{0}-C_{e}\right)V}{m}$$

where *R* is the removal percentage of Hg^2+^ (%); *C*_0_ and *C_e_* refer to the initial and equilibrium Hg^2+^ concentrations (mg L^−1^), respectively; *q_e_* is the removal capacity of Hg^2+^ at equilibrium (mg g^−1^); *V* is the volume of the solution (mL), and m is the weight of BPS (mg). All experiments were conducted in triplicate, and mean values are reported.

Following the batch experiments, the surface morphology of the BPS and the distribution map of Hg^2+^ were examined using a scanning electron microscope equipped with an energy-dispersive X-ray spectrometer (FE-SEM, S-4800, Hitachi, Tokyo, Japan) at 5 kV accelerating voltage, with a working distance of 15 mm. Also, the chemical state of elements on the BPS surface was analyzed via X-ray photoelectron spectroscopy (XPS, K Alpha+, Thermo Scientific, Loughborough, UK) using an Al K α X-ray source.

### 2.3. Hg Adsorption Isotherm

The adsorption capacity of the Hg^2+^ on BPS was analyzed using the Langmuir (see Equation (5)) and Freundlich (see Equation (6)) isotherm models.

(5) $$q_{e}=\frac{Q_{m}bC_{e}}{1+bC_{e}}$$

(6) $$q_{e}=K_{f}C_{e}^{\frac{1}{n}}$$

where *q_e_* is the amount of adsorbed Hg^2+^ at equilibrium (mg g^−1^); *C_e_* is the equilibrium concentration of Hg^2+^ (mg L^−1^) in solution; and *Q_m_* and *b* are the maximum adsorption capacity (mg g^−1^) and Langmuir constant (L mg^−1^) related to free energy of adsorption, respectively. *K_f_* is a constant (mg g^−1^) related to the adsorption capacity and intensity of the Freundlich model, and 1/*n* is the Freundlich constant (unitless) related to the surface heterogeneity. The goodness of fit of these models to Hg adsorption onto BPS was evaluated based on a higher coefficient of determination (r^2^) and a lower standard error.

### 2.4. Hg Adsorption Kinetics

The adsorption kinetics of Hg^2+^ onto BPS were evaluated using pseudo-first order, pseudo-second order, and double-exponential models. The linear form of the pseudo-first order kinetic model is given as Equation (7) [26]:

(7) $$\ln\left(q_{e}-q_{t}\right)=\ln q_{e}-K_{1}t$$

where *q_e_* (mg g^−1^) and *q_t_* (mg g^−1^) are the amounts of Hg^2+^ adsorbed at equilibrium and time *t* (min), respectively. *K*_1_ (min^−1^) is the rate constant of the pseudo-first-order model.

The linear form of the pseudo-second-order kinetic model is shown as Equation (8) [26]:

(8) $$\frac{t}{q_{t}}=\frac{1}{K_{2}q_{e}^{2}}+\frac{t}{q_{e}}$$

where *K*_2_ (g mg^−1^ min^−1^) is the rate constant of the pseudo-second-order model.

The nonlinear form of the double-exponential kinetic model is given as Equation (9) [27]:

(9) $$q_{t}=q_{e}-\frac{D_{1}}{m_{ads}}\exp\left(-K_{D1}t\right)-\frac{D_{2}}{m_{ads}}exp(-K_{D2}t)$$

where *m_ads_* (g L^−1^) is the adsorbent amount in the solution; *D*_1_ and *D*_2_ are adsorption rate constants (g L^−1^) of the rapid and the slow step, respectively; and *K_D_*_1_ and *K_D_*_2_ (min^−1^) are the rate constants of the double exponential model for the fast and slow steps, respectively.

The best fit of Hg adsorption onto BPS using the above three models was evaluated based on a high coefficient of determination (r^2^) and a low root mean square error (RMSE) (see Equation (10)).

(10) $$RMSE=\sqrt{\left(\frac{1}{N-2}\right)\sum_{i=1}^{n}{\left(q_{i,\;exp}-q_{i,cal}\right)}^{2}}$$

where *q_i,exp_* and *q_i,cal_* are the experimental and calculated values of the adsorption capacity, respectively, and *N* is the number of observations in the experiment.

### 2.5. Pseudo-Thermodynamic Parameters of Hg Adsorption

Pseudo-thermodynamic parameters were calculated by conducting batch experiments at 288, 298, and 308 K under the various conditions specified in Table 1. The change in Gibbs free energy of activation ($\Delta G^{o⧧}$) was calculated using the Gibbs–Helmholtz equation (see Equation (11)). By plotting *ln*(*K_e_*) vs. 1/*T* (van’t Hoff equation: see Equation (12)), the change in enthalpy of activation ($\Delta H^{o⧧}$) and the change in entropy of activation ($\Delta S^{o⧧}$) were determined from the slope and intercept of the linear relation, respectively.

(11) $$\Delta G^{o⧧}=\Delta H^{o⧧}-T\Delta S^{o⧧}=-RTlnK_{e}$$

(12) $$lnK_{e}=\frac{\Delta S^{o⧧}}{R}-\frac{\Delta H^{o⧧}}{RT}$$

where *R* is the ideal gas constant (8.314 J∙mol^−1^∙K^−1^); *T* is the absolute temperature (K); and *K_e_* is the binding constant (L g^−1^), which is derived from the Langmuir constant (L mg^−1^) and from the adsorption isotherms [28,29].

### 2.6. Hg Desorption

Desorption studies were conducted using batch conditions similar to those of the adsorption study (Table 1). After Hg was adsorbed onto BPS under specific conditions (adsorbent dose of 1 g L^−1^, initial concentration of Hg 300 mg L^−1^, pH 5, contact time of 24 h, and temperature 298 K), the BPS was separated by filtration and dried. Subsequently, the dried BPS was mixed with desorbing solution using different acids (HCl, HNO_3_, or H_2_SO_4_) at varying molarities (0.1 M, 0.5 M, and 1 M) and shaken for 24 h at a temperature of 298 K. The batch was then filtered through a 0.45 µm membrane filter. The Hg^2+^ concentrations in the filtrate were measured using the cold-vapor AAS Hg analyzer (Hydra II AA, Teledyne Leeman Labs, Hudson, NH, USA). The desorption percentage of Hg^2+^ (DES(Hg), %) was calculated according to the following Equation (13):

(13) $$DES\left(Hg\right)(\%)=\frac{Amount\;of\;desrobed\;{Hg}^{2+}\;into\;the\;desorption\;solution}{Amount\;of\;adsorbed\;{Hg}^{2+}}$$

### 2.7. Hg Removal from Waste Water

Wastewater was collected from a Zn plating plant located in Daegu, Korea, and filtered through a 0.45 µm membrane filter to determine the concentrations of Hg and other metals. Mercury and other heavy metals (As, Cd, Cr, Cu, Ni, Pb, and Zn) co-existed in the collected wastewater (Table 2). Concentrations of Hg in wastewater (0.13 mg L^−1^) exceeded the allowable limit (5 μg L^−1^) for wastewater discharges from the individual industry, as specified by the Korea Water Environment Conservation Act [30]. Because the concentration of Hg was relatively low as compared to other metals, Hg-spiked wastewater was additionally prepared using the same wastewater to compare the Hg removal efficiency. The wastewater was spiked with Hg using HgCl_2_ to prepare a final Hg concentration of 1.5 mg/L. The BPS was added to both actual and spiked wastewaters at a 1 g L^−1^ batch ratio, and the mixture was shaken at 150 rpm at 298 K for 60 min to evaluate the Hg^2+^ removal efficiency using Equation (3). The Hg removal efficiencies of BPS from actual wastewater and spiked wastewater were comparatively assessed.

## 3. Results and Discussion

### 3.1. Biologically Produced S Characteristics

**Figure 1:**
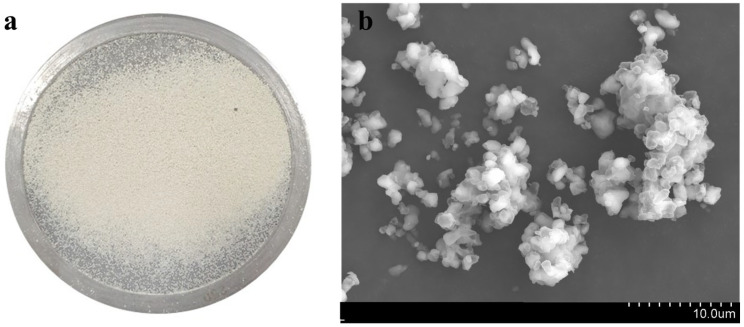
Photo (**a**) and SEM image (**b**) of the biologically produced sulfur (BPS).

The BPS samples collected from the landfill gas desulfurization plant exhibited a light yellow color (Figure 1a) with a slight odor of sulfide. The scanning electron micrographs (SEM) of the BPS (Figure 1b) depicted an amorphous structure composed of spherical sulfur globules with diameters of 10~20 μm. According to Janssen et al. [31], BPS particles are often covered with a negatively charged polymeric protein layer, which could render the particles hydrophilic, despite elemental sulfur being inherently hydrophobic [32,33]. The point of zero charge of BPS was found to be 2.3.

XRF analysis revealed that BPS was composed of various elements, with S being the dominant one, constituting 76% of the composition (Table 3). The elemental composition of BPS used in this experiment was found to be similar to that reported in a previous study [23], despite BPS samples being collected at different times. This suggested that BPS generated from the landfill gas desulfurization plant has a consistent composition.

**Figure 2:**
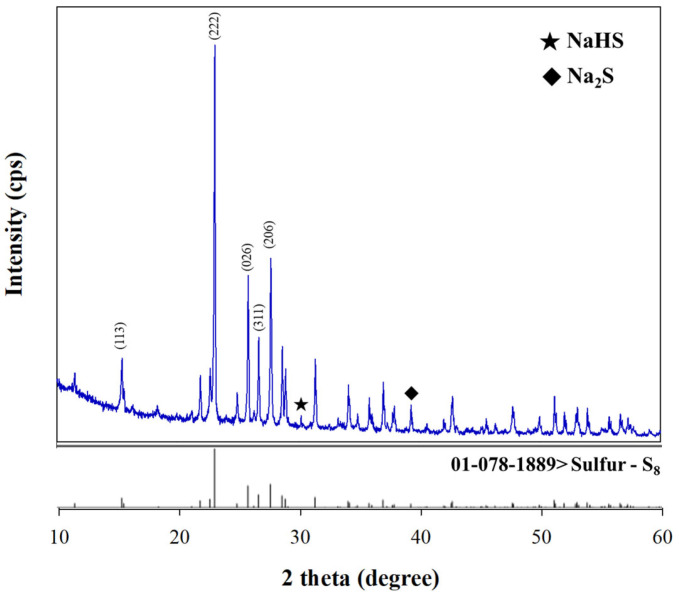
X-ray diffractograms of BPS (**above**) and standard S_8_ reference powder (**below**).

The spectra of X-ray diffractometry (XRD) for BPS powder are depicted in Figure 2. The peaks at 2θ values of 15.38°, 23.07°, 25.83°, 26.71°, and 27.70° were assigned to (113), (222), (026), (311), and (206) reflections of S_8_ (Reference No. 01-078-1889), respectively, while the weak diffraction peaks of BPS corresponded to inorganic sulfides, such as Na_2_S and NaHS [34,35]. The XRD pattern confirmed that BPS was composed of elemental sulfur (S_8_) and sulfides, supporting its strong potential for use as an adsorbent for Hg removal through adsorption processes between Hg- and S-containing ligands. The distribution of BPS particles is relatively broad, with the standard percentiles for particle size D10, D50, and D90 values being 7.4 μm, 125 μm, and 488 μm, respectively. D10, D50, and D90 represent particle sizes at 10%, 50%, and 90% in the cumulative size distribution, respectively. Surface areas of the BPS sample were estimated to be 1.36 m^2^ g^−1^.

### 3.2. Effect of pH and Adsorbent Dose on Hg Removal

**Figure 3:**
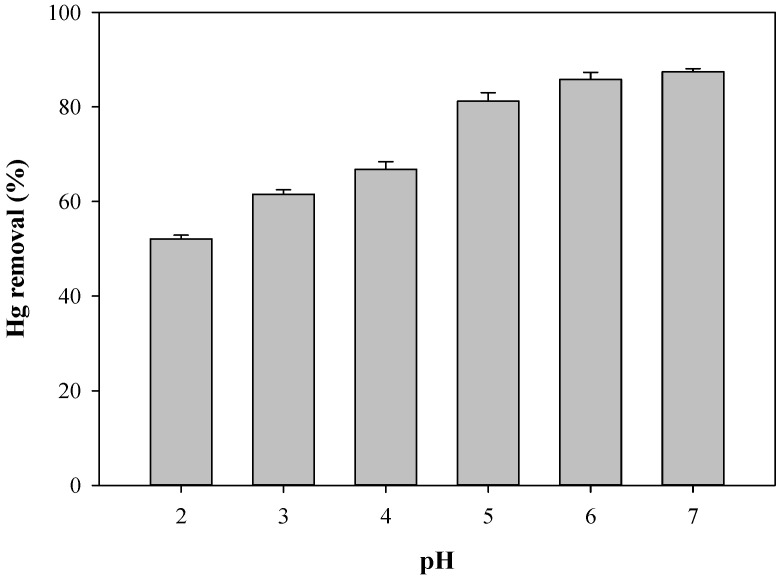
Effect of pH on the removal of Hg^2+^ by BPS. Vertical bars represent standard deviations from the mean values (*n* = 3).

The pH of the batch solution significantly influences the adsorption process, because it affects the surface charge of the adsorbent and the ionization degree and speciation of the adsorbate [36]. To assess the effect of the pH of Hg^2+^ adsorption onto BPS, the initial solution pH values were adjusted to pH 2.0~7.0. The percentage of Hg^2+^ removal by BPS was pH-dependent, showing a sharp increase with increasing pH from 2.0 to 6.0 (Figure 3), followed by a gradual increase at pH values higher than 6. Under strong acidic conditions, high concentrations of H^+^ would compete with Hg^2+^ for adsorption sites on the BPS surface, leading to low Hg^2+^ removal efficiency [37]. Under higher pH conditions, more S^2−^ dissociated from sulfide (NaHS) could favorably complex with free Hg^2+^ to produce HgS precipitates [38]. Additionally, the presence of OH^−^ at a higher pH would facilitate the transformation of Hg^2+^ to Hg(OH)^+^ or Hg(OH)_2_ precipitates [39].

### 3.3. Adsorption Isotherms

**Figure 4:**
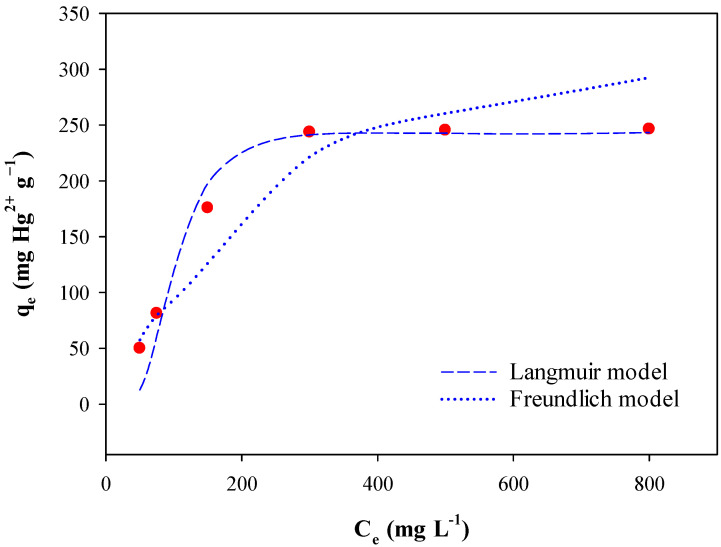
Langmuir and Freundlich isotherms for Hg^2+^ adsorption onto BPS.

The Langmuir and Freundlich isotherm models were employed to evaluate Hg adsorption onto BPS (Figure 4). Table 4 presents the adsorption parameters obtained from both isotherm models. The results of this study confirmed that the Langmuir isotherm model was more applicable, based on a higher coefficient determination (r^2^), than the Freundlich isotherm model in describing the adsorption of Hg^2+^ by BPS. This suggests that Hg adsorption onto BPS occurred uniformly on the finite monolayer sorption sites of BPS. The maximum Hg adsorption capacity (*Q_m_*) of BPS was found to be 244 mg g^−1^. The *Q_m_* values of BPS were compatible to, or even higher than, those reported previously (Table 5), where *Q_m_* values were assessed using various adsorbents, such as activated carbon, functional polymers, and bentonite by-products. These results demonstrate that BPS can be recycled as an effective adsorbent for removing Hg from wastewater.

### 3.4. Adsorption Kinetics

**Figure 5:**
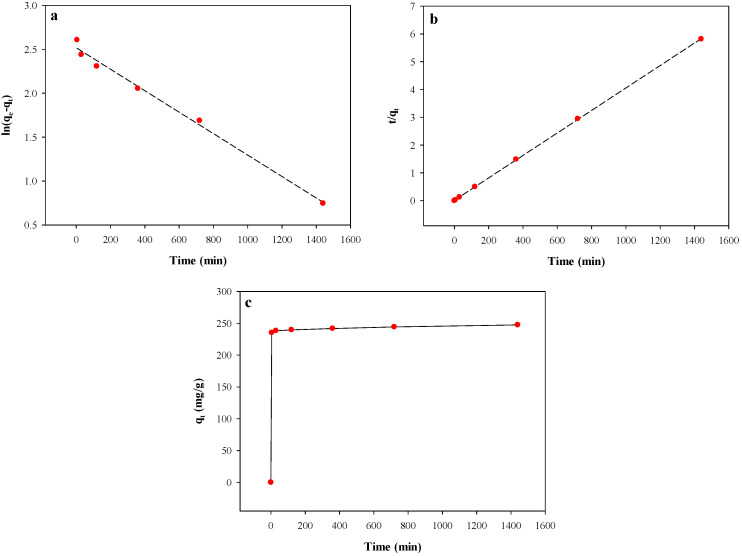
Rate curves of three kinetic models for Hg^2+^ adsorption onto BPS: (**a**) pseudo-first-order kinetic, (**b**) pseudo-second-order, and (**c**) non-linear multiple first-order kinetic model.

Three kinetic models, i.e., the pseudo-first order, pseudo-second order, and the double-exponential models, were employed to investigate the mechanism of Hg adsorption onto BPS. Figure 5 illustrates the rate curves of the three models, and Table 6 summarizes the relevant kinetic parameters. Based on higher r^2^ and lower root mean square error (RMSE) values, the double-exponential first-order kinetic model was found to be the best fit for the Hg adsorption process, even though the other two models showed higher r^2^, as well as higher RSME values.

The Hg adsorption process appears to involve two stages: a fast initial stage followed by a slow stage. The fast adsorption occurred within 5 min, with 75% of Hg removed, followed by a slower and more static adsorption phase. The rate constants of *D*_1_ and *K_D_*_1_ for the fast step of the double-exponential first order kinetic model were 237.2 g L^−1^ and 4.9 min^−1^, respectively, significantly higher than *D*_2_ (11.72 g L^−1^) and *K_D_*_2_ (0.0015 min^−1^) for the slow step. The rate constants for the pseudo-first order kinetic model (*K*_1_) and pseudo-second order kinetic model (*K*_2_) were very low, with values of 0.0012 min^−1^ and 0.0013 g mg^−1^min^−1^, respectively.

The initial fast adsorption process could be interpreted as an adsorption reaction, where Hg species (soft Lewis acid) rapidly complexed with the sulfide functional groups (soft Lewis base) on the BPS surface [31,54]. Studies by Molavi et al. [55] and Li et al. [56] suggest that a greater interaction between adsorbents with high surface area and Hg could contribute to the fast adsorption kinetics. Once rapid adsorption occurs, then the Hg removal efficiency becomes relatively constant even with longer contact times due to the saturation of active sites on the adsorbent surface [23].

### 3.5. Adsorption Thermodynamics

The pseudo-thermodynamic parameters for Hg adsorption onto BPS, including the Gibbs free energy of activation ($\Delta G^{o⧧}$), enthalpy of activation ($\Delta H^{o⧧}$), and entropy of activation ($\Delta S^{o⧧}$), were calculated using the Gibbs–Helmholtz equation (see Equation (11)) and the van’t Hoff equation (see Equation (12)) based on results obtained from the isothermal batch adsorption experiment at different temperatures ranging from 288 to 308 K (Table 7).

As shown in Table 7, the adsorption process of Hg^2+^ onto BPS was spontaneous, as evidenced by the negative value of $\Delta G^{o⧧}$, and endothermic, as indicated by the positive value of $\Delta H^{o⧧}$. Additionally, the positive value of $\Delta S^{o⧧}$ suggests increased disorder and randomness at the solid–liquid interface, which is considered to be a favorable condition during the adsorption process. Results from thermodynamic parameters (Table 7) supported the fact that Hg adsorption onto BPS was a thermodynamically favorable process, because adsorption proportionally increased with increasing temperatures.

### 3.6. Desorption of Hg from HgS Complex

**Figure 6:**
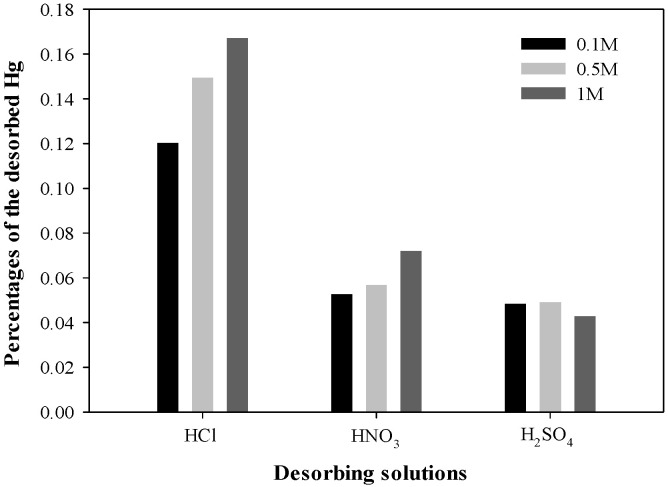
Effect of kind and concentration of acid solutions on the desorption of Hg^2+^ from BPS-adsorbed Hg.

The stability of the adsorbed Hg on BPS was assessed by desorption tests using strong acids, such as HCl, HNO_3_, and H_2_SO_4_, at different ionic strengths ranging from 0.1 to 1.0 M (Figure 6). The percentages of desorbed Hg were highest using HCl, followed by HNO_3_ and H_2_SO_4_. However, the percentages of the desorbed Hg by HCl were lower than 0.17% of the adsorbed Hg, even at 1 M ionic strength. When the ionic strength of the HCl and HNO_3_ solutions increased from 0.1 to 1 M, the amount of desorbed Hg^2+^ increased, but that by H_2_SO_4_ remained relatively constant (Figure 6). These results demonstrate that Hg^2+^ was strongly and irreversibly adsorbed on the BPS due to the high affinity of sulfide anions in BPS (soft Lewis base) towards the Hg^2+^ ion (the soft Lewis acid), according to the HSAB theory [57]. Additionally, the results support the observation that the adsorbed Hg onto BPS would not be released under natural conditions, thereby preventing secondary pollution.

### 3.7. BPS Surface Morphology after Hg Adsorption

**Figure 7:**
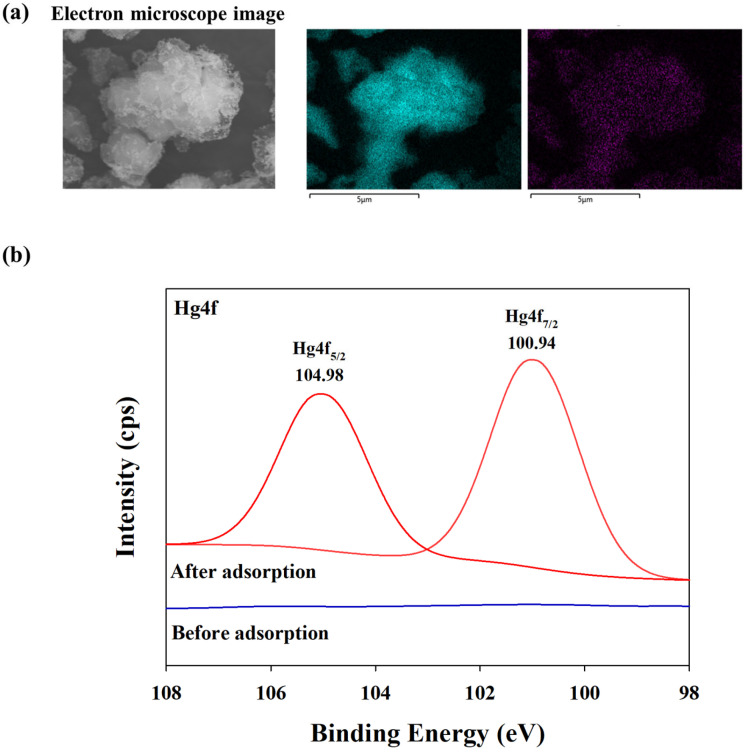
Scanning electron microscope images of BPS, with EDS mapping of S and Hg elements (**a**) and high-resolution XPS spectra of BPS after Hg adsorption (**b**).

To elucidate the mechanism of Hg^2+^ adsorption onto BPS, SEM images and XPS spectra were employed to observe the changes in the BPS surface after Hg adsorption (Figure 7). The SEM images (Figure 7a) revealed that numerous fine particles were spiked onto the surface of BPS after Hg adsorption, indicating the attachment of Hg^2+^ onto the BPS surface. Furthermore, the XPS pattern analysis of Hg4f confirmed that a certain amount of Hg^2+^ was adsorbed onto BPS (Figure 7b). Hg4f refers to the photoelectrons ejected from the 4f orbital of Hg atoms in a sample. The 4f orbital of Hg atom splits into two spin-orbit components when it reacts with X-ray, i.e., 4f5/2 and 4f7/2 [58,59,60]. The Hg4f binding energies for BPS after Hg^2+^ adsorption were mainly centered at 100.94 and 104.98 eV, suggesting that the Hg species adsorbed onto BPS was HgS [61,62]. Therefore, these results demonstrate that Hg^2+^ adsorption onto BPS was mainly governed by chemical complexation on the outer sphere of the BPS surface to form HgS.

### 3.8. Application of BPS for Wastewater Treatment

**Figure 8:**
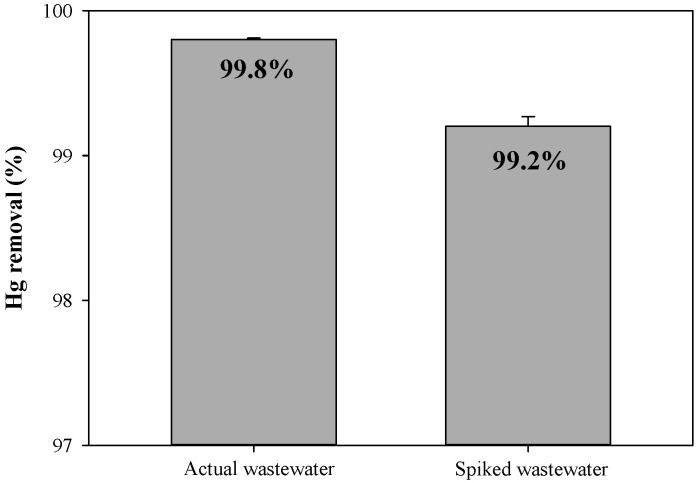
Removal efficiency (%) of Hg^2+^ by BPS from actual and spiked wastewater. Vertical bars represent standard deviations from the mean values (*n* = 3).

The effectiveness of BPS in removing Hg^2+^ from both actual wastewater and spiked wastewater was evaluated to assess its performance under realistic conditions. As depicted in Figure 8, the Hg^2+^ removal percentages by BPS from actual and spiked wastewaters reached 99.8% and 99.2%, respectively, even in the presence of various competing metal ions. These results indicate that the residual concentration of Hg^2+^ in actual wastewater was 0.2 μg L^−1^, which falls within the permissible limit for Hg^2+^ (Table 2). These results may be attributed to the presence of sulfide functional groups in BPS, which are responsible for the preferential adsorption of Hg^2+^ over other co-existing metal ions [63,64]. Additionally, Košak et al. [65] reported that silica nanoparticles containing sulfur as mercapto (–SH) functional groups exhibited the strongest affinity for Hg^2+^ ions (99.9%), followed by Pb^2+^ (55.9%), Cd^2+^ (50.2%), and Zn^2+^ (4%). Consequently, these findings suggest that BPS could serve as a promising adsorbent for the removal of Hg^2+^ from wastewater.

## 4. Conclusions

BPS from the landfill gas desulfurization process was shown to have an amorphous structure made up of micro-sized spherical sulfur globules, where sulfur and sulfide functional groups were enriched. It adsorbed Hg through chemical complexation onto its the monolayer sites, forming a stable HgS complex, which showed a high adsorption capacity of 244 mg g^−1^. This adsorption capacity exceeds that of conventional adsorbents like activated carbon, as reported in the literature. BPS’s adsorption kinetics were rapid, removing 75% of Hg within 5 min. HgS complex formation was an irreversible and thermodynamically favorable process, with a spontaneous and endothermic reaction. BPS demonstrated a preference for Hg removal, achieving over 99% efficiency, even in the presence of other co-existing metals in wastewater. These results show the potential of BPS as a cost-effective and environmentally friendly adsorbent for treating wastewater, specifically wastewater containing Hg, with promising prospects for recycling.

## Figures and Tables

**Table 1:** Descriptions of conditions of the batch adsorption experiments.

	pH	Adsorbent Dose (g L^−1^)	Contact Time (min)	Initial Hg^2+^ Concentration (mg L^−1^)	Temperature (K)
Effect of pH	2, 3, 4, 5, 6 and 7	1	1440	300	298
Effect of time	5	1	5, 30, 120, 360, 720, 1440	300	298
Effect of Hg^2+^ concentration	5	1	1440	50, 150, 300, 500, 800	288, 298, 308

**Table 2:** Metal concentrations of wastewater sample used in this study and allowable limit of each metal in discharging water.

	As	Cd	Cr	Cu	Pb	Zn	Hg
Wastewater (mg L^−1^)	4.2	118	0.61	49	1.45	22,727	0.13
Allowable limit ^†^ (mg L^−1^)	0.25	0.1	2	3	0.5	5	0.005

^†^ Allowable limit for wastewater discharges from the individual industry specified by the Korea Water Environment Conservation Act.

**Table 3:** Elemental composition of biologically produced S (BPS) determined by X-ray fluorescence spectrometry.

	S	O	Na	C	K	Si	P
BPS (% by mass)	76.1	15.9	5.55	2.41	0.02	0.01	0.01

**Table 4:** Adsorption parameters of the Langmuir and Freundlich isotherm models.

	Langmuir Model ^†^			Freundlich Model		
	*Q_max_* (mg g^−1^)	*b* (L mg^−1^)	r^2^	*K_f_* (mg g^−1^)	*n*	r^2^
BPS	243.9	0.56	0.99 **	87.3	5.52	0.92 **

** Significant at *p* < 0.001. ^†^ Refer to Equations (5) and (6) for parameter descriptions.

**Table 5:** Lists of maximum capacities (*Q_max_*) for Hg^2+^ adsorption by various adsorbents reported in selected literature.

Adsorbents	Temperature (°C)	Dose (g/L)	Concentration (mg/L)	pH	*Q_max_* (mg/g)	r^2^	References
Activated carbon (from mango seed) activated with CaCl_2_ or H_2_SO_4_	Room	3.33	10–150	5.0	74.45 79.11	0.905 0.903	[40]
Activated carbon (from mango seed) activated with CaCl_2_ or H_2_SO_4_ and functionalized with Na_2_S	Room	3.33	10–150	5.0	92.16 124.13	0.925 0.910	[40]
Activated carbon (from furfural) activated with steam	Room	0.2	10–40	5.5	174	-	[41]
Mesoporous silica functionalized with propylthiol	20 °C	0.57	30–600	-	110.32–577.70	-	[42]
Activated carbon (from walnut shell) activated with ZnCl_2_	29 °C	1	9.7–107	5.0	100.9 151.5	0.998 0.999	[43]
Chitosan beads grafted with polyacrylamide	Room	0.25	10–200	4.0	322.6	0.997	[44]
Bentonite modified with mercapto	37.28 °C	1.9	5–40	6.17	32.89	0.99	[45]
Ti_3_C_2_T_x_ MXene functionalized with thioacetamnide and sodium molybdate	-	1	50–2000	6.5	1446.26	0.984	[46]
Activated carbon (from coir pith)	Room	0.2	10–40	5.0	154	-	[47]
Activated carbon (from *Ceiba pentandra* hulls)	30 °C	-	10–140	6.0	25.88	0.8167	[48]
Activated carbon (from *Phaseolus aureus* hulls)	30 °C	-	10–140	7.0	23.66	0.9016	[48]
Activated carbon (from *Cicer arietinum* waste)	30 °C	-	10–140	7.0	22.88	0.9273	[48]
Zeolitized coal fly ash	Room	10–100	10	2.5	0.44	0.96	[19]
Porous sulfur copolymer	25 °C	0.1	2–10	-	0.37	0.999	[49]
Desiccated coconut waste	30 °C	1	25–500	7.4	500	0.970	[50]
Activated carbon (from fruit shell of *Terminalia catappa* L.) activated with H_2_SO_4_	32 °C	0.05–5.0	30	5.0	94.43	0.9956	[51]
Tree fern	10–25 °C	5	55–145	-	20.2–26.5	-	[52]
Exhausted coffee waste	33 °C	4	50–110	7.0	31.75	0.99	[53]

**Table 6:** Kinetic model parameters for Hg^2+^ adsorption onto BPS.

Kinetic Models	Parameters ^†^	Values
Pseudo-first-order	*q_e_* (mg g^−1^)	12.41
	*K_1_* (min^−1^)	0.0012
	r^2^	0.99 **
	RMSE	394.73
Pseudo-second-order	*q_e_* (mg g^−1^)	250.00
	*K_2_* (g mg^−1^min^−1^)	0.0013
	r^2^	0.99 **
	RMSE	31.54
Double-exponential	*q_e_* (mg g^−1^)	248.89
	*D_1_* (g L^−1^)	237.17
	*K_D1_* (min^−1^)	4.9438
	*D_2_* (g L^−1^)	11.72
	*K_D2_* (min^−1^)	0.0015
	r^2^	0.99 **
	RMSE	0.63

^†^ Refer to Equations (7)–(10) for parameter descriptions. ** Significant at *p* < 0.001.

**Table 7:** Thermodynamic parameters for adsorption of Hg^2+^ onto BPS.

	Temperature (K)	$\Delta G^{o⧧}$ (kJ mol^−1^)	$\Delta H^{o⧧}$ (kJ mol^−1^)	$\Delta S^{o⧧}$ (J mol^−1^ K^−1^)
BPS	288	−12.6	61.2	256.7
	298	−15.7		
	308	−17.7		

## Data Availability

The data presented in this study are available upon request from the corresponding author.

## References

[1] World Health Organization Mercury and Health. https://www.who.int/news-room/fact-sheets/detail/mercury-and-health.

[2] Counter S.A., Buchanan L.H. (2004). Mercury exposure in children: A review. Toxicol. Appl. Pharmacol..

[3] Rice K.M., Walker E.M., Wu M., Gillette C., Blough E.R. (2014). Environmental mercury and its toxic effects. J. Prev. Med. Public Health.

[4] Holmes P., James K.A.F., Levy L.S. (2009). Is low-level environmental mercury exposure of concern to human health?. Sci. Total Environ..

[5] Paduraru E., Iacob D., Rarinca V., Rusu A., Jijie R., Ilie O.D., Ciobica A., Nicoara M., Doroftei B. (2022). Comprehensive review regarding mercury poisoning and its complex involvement in Alzheimer’s disease. Int. J. Mol. Sci..

[6] Sarkar S., Gill S.S., Gupta G.D., Verma S.K. (2022). Water toxicants: A comprehension on their health concerns, detection, and remediation. Environ. Sci. Pollut. Res..

[7] Naija A., Yalcin H.C. (2023). Evaluation of cadmium and mercury on cardiovascular and neurological systems: Effects on humans and fish. Toxicol. Rep..

[8] Albatrni H., Qiblawey H., El-Naas M.H. (2021). Comparative study between adsorption and membrane technologies for the removal of mercury. Separ. Purif. Technol..

[9] Sharma A., Sharma A., Arya R.K. (2015). Removal of mercury (II) from aqueous solution: A review of recent work. Separ. Sci. Technol..

[10] Feng Q., Yang W., Chang M., Wen S., Liu D., Han G. (2024). Advances in depressants for flotation separation of Cu-Fe sulfide minerals at low alkalinity: A critical review. Int. J. Miner. Metall. Mater..

[11] Feng Q., Zhang G., Zhang Q., Zhao W. (2024). Synergistic activation of sulfidized hemimorphite with copper-lead species for improving surface hydrophobicity and floatability. Sep. Purif. Technol..

[12] Yu J.G., Yue B.Y., Wu X.W., Liu Q., Jiao F.P., Jiang X.Y., Chen X.Q. (2016). Removal of mercury by adsorption: A review. Environ. Sci. Pollut. Res..

[13] Liu Z., Sun Y., Xu X., Qu J., Qu B. (2020). Adsorption of Hg(II) in an aqueous solution by activated carbon prepared from rice husk using KOH activation. ACS Omega.

[14] Das R., Giri S., Muliwa A.M., Maity A. (2017). High-performance Hg(II) removal using thiol-functionalized polypyrrole (PPy/MAA) composite and effective catalytic activity of Hg(II)-adsorbed waste material. ACS Sustain. Chem. Eng..

[15] Velempini T., Pillay K. (2019). Sulphur functionalized materials for Hg(II) adsorption: A review. J. Environ. Chem. Eng..

[16] Qiu Y., Zhang Z., Zhang T., Zhang P. (2022). Sulfide modifies physicochemical properties and mercury adsorption of microplastics. Sci. Total Environ..

[17] Huang L., Shen R., Liu R., Shuai Q. (2020). Thiol-functionalized magnetic covalent organic frameworks by a cutting strategy for efficient removal of Hg^2+^ from water. J. Hazard. Mater..

[18] Xu D., Wu W.D., Zi H.J., Yang R.X., Deng W.Q. (2018). Sulfur rich microporous polymer enables rapid and efficient removal of mercury(II) from water. Chemosphere.

[19] Attari M., Bukhari S.S., Kazemian H., Rohani S. (2017). A low-cost adsorbent from coal fly ash for mercury removal from industrial wastewater. J. Environ. Chem. Eng..

[20] Ochedi F.O., Liu Y., Hussain A. (2020). A review on coal fly ash-based adsorbents for mercury and arsenic removal. J. Clean. Prod..

[21] Wang L., Hou D., Cao Y., Ok Y.S., Tack F.M.G., Rinklebe J., O’Connor D. (2020). Remediation of mercury contaminated soil, water, and air: A review of emerging materials and innovative technologies. Environ. Int..

[22] Hu L., Du Y., Long Y. (2017). Relationship between H_2_S emissions and the migration of sulfur-containing compounds in landfill sites. Ecol. Eng..

[23] Kim H.S., Jeong S.S., Lee J.G., Yoon J.H., Lee S.P., Kim K.R., Kim S.C., Kirkham M.B., Yang J.E. (2021). Biologically produced sulfur as a novel adsorbent to remove Cd^2+^ from aqueous solutions. J. Hazard. Mater..

[24] Southern Research Institute (2004). Environmental Technology Verification Report—NATCO Group, Inc.—Paques THIOPAQ Gas Purification Technology.

[25] Heo J., Lee B., Kim S., Kim J.N., Lim H. (2018). Techno-economic analysis of a biological desulfurization process for a landfill gas in Korea. Sep. Sci. Technol..

[26] Shen Y., Li H., Zhu W., Ho S.H., Yuan W., Chen J., Xie Y. (2017). Microalgal-biochar immobilized complex: A novel efficient biosorbent for cadmium removal from aqueous solution. Bioresour. Technol..

[27] Chiron N., Guilet R., Deydier E. (2003). Adsorption of Cu(II) and Pb(II) onto a grafted silica: Isotherms and kinetic models. Water Res..

[28] Georgieva V.G., Gonsalvesh L., Tavlieva M.P. (2020). Thermodynamics and kinetics of the removal of nickel (II) ions from aqueous solutions by biochar adsorbent made from agro-waste walnut shells. J. Mol. Liq..

[29] Venkiteshwaran K., Wells E., Mayer B.K. (2020). Kinetics, affinity, thermodynamics, and selectivity of phosphate removal using immobilized phosphate-binding proteins. Environ. Sci. Technol..

[30] Ministry of Environment Water Environment Conservation Act. https://www.law.go.kr/LSW//lsInfoP.do?lsiSeq=231465&urlMode=engLsInfoR&viewCls=engLsInfoR#0000.

[31] Janssen A.J.H., Lettinga G., de Keizer A. (1999). Removal of hydrogen sulphide from wastewater and waste gases by biological conversion to elemental Sulphur Colloidal and interfacial aspects of biologically produced sulphur particles. Colloids Surf. A Physicochem. Eng. Asp..

[32] Kleinjan W.E., de Keizer A., Janssen A.J.H. (2005). Kinetics of the reaction between dissolved sodium sulfide and biologically produced sulfur. Ind. Eng. Chem. Res..

[33] Kuklińska K., Wolska L., Namieśnik J., Cieszynska M. (2013). Analytical and bio-analytical problems associated with the toxicity of elemental sulfur in the environment. Trends Anal. Chem..

[34] Aranzabe E., Villasante P.M., March R., Arriortua M.I., Larrañaga A., Aranzabe A. (2015). More than color: Pigments with thermal storage capacity; processing and degradation behavior. Adv. Mater. Phys. Chem..

[35] Li X., Morrish R.M., Yang Y., Wolden C.A., Yang Y. (2015). Thermodynamically favorable conversion of hydrogen sulfide into valuable products through reaction with sodium naphthalenide. ChemPlusChem.

[36] Zhang W., Zhang Y., Gutha Y., Xu J. (2017). Adsorption of Pb(II) ions from aqueous environment using eco-friendly chitosan schiff’s base@Fe_3_O_4_ (CSB@Fe_3_O_4_) as an adsorbent; kinetics, isotherm and thermodynamic studies. Int. J. Biol. Macromol..

[37] Fan L., Zhou A., Zhong L., Zhang Z., Liu Y. (2019). Selective and effective adsorption of Hg(II) from aqueous solution over wide pH range by thiol functionalized magnetic carbon nanotubes. Chemosphere.

[38] Tang T., Xu J., Lu R., Wo J., Xu X. (2010). Enhanced Hg^2+^ removal and Hg^0^ re-emission control from wet fuel gas desulfurization liquors with additives. Fuel.

[39] Qu Z., Fang L., Chen D., Xu H., Yan N. (2017). Effective and regenerable Ag/graphene adsorbent for Hg(II) removal from aqueous solution. Fuel.

[40] Caicedo Saceldo O.D., Varga D.P., Giraldo L., Moreno-Piraján J.C. (2021). Study of mercury [Hg(II)] adsorption from aqueous solution on functionalized activated carbon. ACS Omega.

[41] Yardim M.F., Budinova T., Ekinci E., Petrove N., Razvigorova M., Minkova V. (2003). Removal of mercury (II) from aqueous solution by activated carbon obtained from furfural. Chemosphere.

[42] Aguado J., Arsuaga J.M., Arencibia A. (2005). Adsorption of aqueous mercury(II) on propylthiol-functionalized mesoporous silica obtained by cocondensation. Ind. Eng. Chem. Res..

[43] Zabihi M., Asl A.H., Ahmadpour A. (2010). Studies on adsorption of mercury from aqueous solution on activated carbons prepared from walnut shell. J. Hazard. Mater..

[44] Li N., Bai R., Liu C. (2005). Enhanced and selective adsorption of mercury ions on chitosan beads grafted with polyacrylamide via surface-initiated atom transfer radical polymerization. Langmuir.

[45] Şahan T., Erol F., Yilmaz Ş. (2018). Mercury(II) adsorption by a novel adsorbent mercapto-modified bentonite using ICP-OES and use of response surface methodology for optimization. Microchem. J..

[46] Shahzad A., Jang J., Lim S.R., Lee D.A. (2020). Unique selectivity and rapid uptake of molybdenum-disulfide-functionalized MXene nanocomposite for mercury adsorption. Environ. Res..

[47] Namasivayam C., Kadirvelu K. (1999). Uptake of mercury (II) from wastewater by activated carbon from an unwated agricultural solid by-product: Coirpith. Carbon.

[48] Rao M.M., Reddy D.H.K.K., Venkateswarlu P., Seshaiah K. (2009). Removal of mercury from aqueous solutions using activated carbon prepared from agricultural by-product/waste. J. Environ. Manag..

[49] Wadi V.S., Mittal H., Fosso-Kankeu E., Jena K.K., Alhassan S.M. (2020). Mercury removal by porous sulfur copolymers: Adsorption isotherm and kinetics studies. Colloids Surf. A.

[50] Johari K., Saman N., Song S.T., Mat H., Stuckey D.C. (2013). Utilization of coconut milk processing waste as a low-cost mercury sorbent. Ind. Eng. Chem. Res..

[51] Inbaraj B.S., Sulochana N. (2006). Mercury adsorption on a carbon sorbent derived from fruit shell of *Terminalia catappa*. J. Hazard. Mater..

[52] Ho Y.S., Wang C.C. (2008). Sorption equilibrium of mercury onto ground-up tree fern. J. Hazard. Mater..

[53] Alvarez N.M.M., Pastrana J.M., Lagos Y., Lozada J.J. (2018). Evaluation of mercury (Hg^2+^) adsorption capacity using exhausted coffee waste. Sustain. Chem. Pharm..

[54] Gharabaghi M., Irannajad M., Azadmehr A.R. (2012). Selective sulphide precipitation of heavy metals from acidic polymetallic aqueous solution by thioacetamide. Ind. Eng. Chem. Res..

[55] Molavi H., Hakimian A., Shojaei A., Raeiszadeh M. (2018). Selective dye adsorption by highly water stable metal-organic framework: Long term stability analysis in aqueous media. Appl. Surf. Sci..

[56] Li H., Jin R., Hu H., Kalkhajeh Y.K., Zhao Y., Gao Y., Zhang B. (2021). Adsorption of As(III), Pb(II), and Zn(II) from wastewater by sodium alginate modified materials. J. Anal. Methods Chem..

[57] Asiabi H., Yamini Y., Shamsayei M., Molaei K., Shamsipur M. (2018). Functionalized layered double hydroxide with nitrogen and sulfur co-decorated carbondots for highly selective and efficient removal of soft Hg^2+^ and Ag^+^ ions. J. Hazard. Mater..

[58] Lu P., Chen T., Liu H., Li P., Peng S., Yang Y. (2019). Green preparation of nanoporous pyrrhotite by thermal treatment of pyrite as an effective Hg(II) adsorbent: Performance and mechanism. Minerals.

[59] Wang C., Zhang X., Mei J., Hu Q., Yang S. (2020). Outstanding performance of magnetically separable sulfureted MoO_3_/Fe−Ti spinel for gaseous Hg^0^ recovery from smelting flue gas: Mechanism and adsorption kinetics. Environ. Sci. Technol..

[60] Song S., Li Y., Liu Q.S., Wang H., Li P., Shi J., Hu L., Zhang H., Liu Y., Li K. (2021). Interaction of mercury ion (Hg^2+^) with blood and cytotoxicity attenuation by serum albumin binding. J. Hazard. Mater..

[61] Singh N., Patil K.R., Khanna P.K. (2007). Nano-sized HgSe powder: Single-step preparation and characterization. Mater. Sci. Eng. B.

[62] Duan L., Hu X., Sun D., Liu Y., Guo Q., Zhang T., Zhang B. (2020). Rapid removal of low concentrations of mercury from wastewater using coal gasification slag. Korean J. Chem. Eng..

[63] Fu W., Wang X., Huang Z. (2019). Remarkable reusability of magnetic Fe_3_O_4_-encapsulated C_3_N_3_S_3_ polymer/reduced graphene oxide composite: A highly effective adsorbent for Pb and Hg ions. Sci. Total Environ..

[64] Wang Y., Sun H., Li C., Meng H., Lu Y., Li Y. (2022). A novel Sulfur-functionalized alkynyl carbon material for highly efficient removal of Hg(II) from water. Sep. Purif. Technol..

[65] Košak A., Lobnik A., Bauman M. (2015). Adsorption of mercury (II), lead (II), cadmium (II) and zinc (II) from aqueous solutions using mercapto-modified silica particles. Int. J. Appl. Ceram. Technol..

